# Cognitive Stimulation Therapy Outcomes Across Healthcare and Community Settings for Individuals With Dementia

**DOI:** 10.1093/geroni/igaa057.200

**Published:** 2020-12-16

**Authors:** Randy Gallamore

**Affiliations:** Saint Louis University, St. Louis, Missouri, United States

## Abstract

Cognitive Stimulation Therapy (CST) is an evidenced-based intervention for individuals with mild to moderate dementia. Originally developed in the U.K., CST has been adapted in several areas of the United States as a meaningful group intervention to help aid in recall and reminiscence for this population. Adaptations of CST have now been developed, including the application of these groups in medical and healthcare settings. However, no study to date has compared memory and mood outcomes of community CST groups to healthcare CST groups. This study will examine the differences in memory, mood and physical mobility scores across both rural and urban settings where CST is used. Two-hundred and sixty-six total participants who have completed all 14 sessions were analyzed, with 150 who participated in a rural hospital and 116 who were in community or university settings. Preliminary data shows that CST is an intervention that can be used effectively in both environments. The results from this study show that improvements in scores were seen in both community (SLUM = +1.75; Cornell = -1.41) and healthcare settings (SLUM = +2.59; Cornell = -2.63). CST might be a meaningful intervention to also help in decrease depression and loneliness in this population. Continued group interventions should be developed in medical and healthcare settings as a resource for patients and family members with dementia-related disorders. There should also be further consideration on the factors that impacted the difference between the two settings.

